# High intensity lifestyle intervention and long-term impact on weight and clinical outcomes

**DOI:** 10.1371/journal.pone.0195794

**Published:** 2018-04-18

**Authors:** Linda Gotthelf, Ya-Ting Chen, Srinivasan Rajagopalan, Elise Chi-Tao Wu, Ishita Doshi, Carol Addy

**Affiliations:** 1 HMR Weight Management Services Corp., Boston, Massachusetts, United States of America; 2 Center for Outcomes Research and Real-World Evidence, Healthcare Services and Solutions, Merck & Co., Inc, Kenilworth, New Jersey, United States of America; 3 Med Data Analytics, Inc., Milltown, New Jersey, United States of America; 4 Center for Outcomes Research and Real-World Evidence, Economics and Data Science, Merck & Co., Inc, Kenilworth, New Jersey, United States of America; 5 Center for Outcomes Research and Real-World Evidence, Study Management, Merck & Co., Inc, Kenilworth, New Jersey, United States of America; Weill Cornell Medical College in Qatar, QATAR

## Abstract

**Background:**

Obesity increases the risk for diabetes and cardiovascular events, with a corresponding growth in medical costs. High intensity lifestyle intervention (HILI) is the cornerstone for weight management. We assessed the effectiveness of clinic-based HILI on weight loss and associated clinical outcomes by duration of program participation and comorbid conditions.

**Methods:**

This was a retrospective cohort study of patients who enrolled in HILI weight management programs at Health Management Resources (HMR) clinics located across the U.S. Patients completed health risk assessments (HRA) and were enrolled for up to 24 months at the time of follow-up HRA. HMR programs provide weekly group coaching to achieve reduced calorie intake, increased fruit/vegetable intake, and physical activity ≥2,000 kcal/wk. A Markov model predicted avoidance of diabetes and cardiovascular events and projected cost savings due to weight loss.

**Results:**

Of the 500 patients included in the analysis, 67% were female and mean age was 54.1 years (s.d. 11.6). The baseline weight and BMI were 243.5 lbs (range 144.0–545.0) and 38.8 kg/m2 (range 25.4–85.0), respectively. Overall, patients lost an average of 47.4 lbs (18.9% of initial body weight [IBW]); the amount of weight loss was consistent among those with diabetes/pre-diabetes (50%), high/moderate risk for dyslipidemia (60%), hypertension/pre-hypertension (86%), and severe obesity (37%). The mean IBW lost was 16.4%, 19.3%, 20.7% for ≤6 months (n = 165), 7–12 months (n = 140), 13–24 months (n = 195) of program participation, respectively. The simulation model estimated 22 diabetes and 30 cardiovascular events and $1,992,370 medical costs avoided over 5 years in the 500 patients evaluated.

**Conclusion:**

Patients in the HMR clinic-based HILI program achieved substantial weight loss regardless of duration of program participation, risk profile and comorbid status. The HMR program could be an effective strategy to prevent costly diabetes and cardiovascular events, particularly in high risk patients.

## Introduction

The most recent data show that more than 70% of Americans are overweight or obese [1], with over one-third (37.9%) being obese [2]. National health expenditures are projected to grow at an average annual rate of 5.6% between 2016 and 2025 and are estimated to represent 19.9% of gross domestic product by 2025 [3]. Despite this worsening economic burden, there are few effective therapeutic options that provide long-term weight loss benefit, including pharmacotherapy or surgical interventions.

Intensive lifestyle intervention is the foundation of effective long-term weight management, as demonstrated with the Diabetes Prevention Program [4] and the Look AHEAD [5] study. Joint guidelines from the American Heart Association, American College of Cardiology, and The Obesity Society published in 2013 assigned strong evidence to comprehensive, high intensity (>14 sessions in 6 months) lifestyle intervention (HILI) and recommended that healthcare professionals “advise overweight and obese individuals who would benefit from weight loss to participate for ≥6 months in a comprehensive lifestyle program that assists participants in adhering to a lower calorie diet and in increasing physical activity through the use of behavioral strategies”, with an initial weight loss goal of 5%-10% of baseline weight within 6 months [6]. These recommendations are closely aligned with those of the American Association of Clinical Endocrinologists and American College of Endocrinology as well as the U.S. Preventive Services Task Force guidelines on obesity management for adults. [7,8]

There are a variety of lifestyle based weight management options available; however, not all are HILI programs, and retention and sustained engagement in many programs is problematic. Furthermore, the effectiveness of such programs in a real world setting that more closely reflects the actual patient experience is rarely reported.

In the current study, we evaluate the real world effectiveness of one clinic-based HILI program (HMR Weight Management Services Corp., Boston, MA). Previous studies evaluating the effectiveness of HMR clinic-based HILI programs for weight loss and improvement in related health risk factors were mostly conducted in relatively small cohorts of patients and with short duration of follow-up [9,10]. We hypothesized that high intensity lifestyle intervention would result in clinically meaningful weight reduction and improved health outcomes. The primary objective in this study was to assess the impact of the HMR clinic-based HILI program on short- and long-term weight management benefit overall and in high risk populations (e.g., patients with diabetes, severe obesity, and hypertension). The secondary objective was to understand the impact of HILI on changes in biometric indices and medication utilization. As an exploratory objective, we used a simulation model to project clinical outcomes avoided and financial implications based upon real world weight loss achieved in the HMR clinic-based HILI program.

## Methods

### Study design and patient population

This was a retrospective cohort study of adult patients who voluntarily enrolled in one of 43 HMR clinic-based weight management programs in locations across the U.S. Patients completed a health risk assessment (HRA) at enrollment (baseline) and a follow-up HRA in July-August 2013 during participation in the weight maintenance phase of the program. Patients had continuous enrollment in the HMR program between the time of enrollment and completion of the HRA in July/August 2013, duration of participation of less than 24 months, and had complete and valid biometric data.

This study was approved by the New England Independent Review Board (Needham, MA, USA) which determined that the study met requirements for a waiver of informed consent based on criteria from 45 CFR 46.116(d).

### Intervention

Clinic-based programs offered by HMR provide intensive behavioral-based intervention to facilitate lifestyle change with the following goals: 1) to increase physical activity (PA); 2) to improve dietary behaviors as achieved by increasing vegetable and fruit intake (V/F) and strategic use of portion-controlled foods (PCF), which are tools to maintain calorie control for weight loss; and 3) to develop behavioral skills to effectively manage the environment and social situations. All of these behaviors are clearly defined enabling patients to keep simple, daily records (using an app or paper), which enhances patient accountability and which also provides the basis for weekly coaching intervention.

Trained HMR coaches help participants to learn new healthy lifestyle skills and how to turn those skills into new healthier habits. In the weekly group classes, participants learn the skills needed to lose weight and to maintain weight loss and benefit from the experience and insight of other participants in the group.

The HMR program is divided into two phases, including Phase 1 (weight-loss) and Phase 2 (weight-maintenance). There was no pre-defined duration of participation for either phase of the program. Patients were encouraged to participate in Phase 1 until they reached their weight loss goal, after which they transitioned to Phase 2. Patients were encouraged to participate in Phase 2 for 12–18 months, however some patients elected to participate for longer than this duration.

Phase 1 (weight loss phase) is designed to allow patients to achieve desired weight loss safely as soon as possible with the assistance of a structured diet using PCFs. Phase 1 also enables participants to begin to learn strategies for changes in diet and PA, which are necessary for long-term weight and health management, as delivered through weekly 75 to 90-minute face-to-face group coaching sessions. Two diet options are offered in clinic-based programs for weight loss: Decision-Free^®^ (using only PCFs) and Healthy Solutions^®^ (PCFs plus V/F). These options vary in terms of calorie level and the potential need for medical supervision.

Phase 2 is the weight maintenance phase of HMR programs. Participants progress to Phase 2 after having reached their goal weight or when they are ready for less structure in their diet. During Phase 2 participants continue to attend weekly 60-minute coaching sessions, where they learn even more strategies to manage their weight as they face “real world” eating challenges such as socializing, dining out, or traveling. Participants are coached in maintaining PA (≥ 2,000 kcal/week) and in making healthier eating choices (lean proteins, whole grains, V/F), with optional/strategic use of PCFs to help with additional weight loss or maintaining weight loss.

### Data source and analysis

Information on family and personal health, current lifestyle risk factors, such as PA, nutrition, alcohol use, and medication use for treatment of hypertension, diabetes, and dyslipidemia, were self-reported using the HRA questionnaire. Physical activity was assessed based on the amount of time that each individual typically engaged in various physical activities per week over the last 6 months, as reported in the HRA: “In the last 6 months, how much time have you spend in a typical week consistently doing activities like those listed below: Moderate Exercise (e.g., walking, golf, calisthenics), Vigorous Exercise (e.g., brisk walking, basketball, racquetball), Very Vigorous Exercise (e.g., running, cycling, stair climbing), Non-Recreational Exercise (e.g., chopping wood, raking, shoveling)?” Responses ranged from less than 15 minutes per week to more than 6 hours per week. Total time and activity intensity, along with the individual’s body weight was used to estimate physical activity calories, taking into consideration greater estimated calorie expenditure for individuals of higher body weight relative to those of lower body weight for the same time and intensity level of activity.

Nutrition was assessed by responses to questions pertaining to percent dietary fat and V/F intake. Patients selected the types of foods typically eaten. Based on the responses, a score of <20% or >50% percent fat diet was given for low- and high-fat diet, respectively. Vegetable/fruit intake was assessed by the number of days per week that the respondent typically eats 2 servings of vegetables and 1 fruit per day. The score ranges between >2 vegetables and 1 fruit daily to <1 day per week. Duration of program participation was the time between baseline HRA and follow-up HRA.

Biometric parameters were assessed by clinic health professionals and included height, weight and blood pressure (BP). Weight was assessed on a weekly basis by the clinic staff generally at the same time of day (e.g., immediately preceding the weekly coaching intervention). Body mass index was calculated using weight (kg) divided by height (m^2^). Obesity severity was classified as: severe obesity (BMI ≥ 40), class II obesity (BMI ≥ 35 and < 40), class I obesity (≥ 30 and < 35) and overweight (BMI < 30). Blood pressure was assessed using the auscultatory method and a properly calibrated and validated instrument. Patients were seated quietly for at least 5 minutes in a chair (rather than an exam table), with feet on the floor, and arm supported at the level of the heart. At least two measurements were obtained. The following laboratory parameters were also assessed: total cholesterol/high-density lipoproteins (TC/HDL), triglycerides (TG), and fasting blood glucose (FBG). Low-density lipoprotein cholesterol (LDL-C) was calculated using the Freidewald equation [LDL cholesterol (mg/dL) = total cholesterol—HDL cholesterol–(triglycerides/5)], where all units are mg/dL. Since this method is valid only for TG values between 100 mg/dL and 400 mg/d, LDL-C values were set to missing for TG values outside of this range.

Baseline comorbid conditions of diabetes, pre-diabetes, hypertension, pre-hypertension, and dyslipidemia were reported based on HRA and biometric values. A patient was identified as having diabetes at baseline if one of the following conditions was satisfied: on oral diabetic medication, on insulin, or baseline FBG > 125 mg/dL[11]. Pre-diabetes was defined as absence of oral diabetic medication or insulin and a baseline FBG between 100mg/dL and 125 mg/dL, both inclusive. Hypertension at baseline was identified if one of the following conditions was satisfied: on oral hypertension medication, baseline systolic BP ≥ 140 mmHg or baseline diastolic BP ≥ 90 mmHg[12]. Pre-hypertension was defined as absence of hypertension medication and, either baseline systolic BP between 120 mmHg and 139 mmHg (both inclusive) or baseline diastolic BP between 80 mmHg and 89 mmHg (both inclusive). Risk for dyslipidemia was defined as high if patient was taking anti-dyslipidemia medication or baseline LDL-C ≥ 160 mg/dL; moderate if no cholesterol medication and baseline LDL-C ≥ 130 mg/dL and < 160 mg/dL; or low if no cholesterol medication and baseline LDL-C < 130 mg/dL[13].

The primary endpoint of the study was weight/BMI reduction; the secondary endpoints were other biometric parameters, including blood pressure, glucose, and lipids, and medication utilization. Descriptive analyses were performed and means and standard deviations were reported for continuous variables and proportions for categorical variables. Statistical significance of observed differences in continuous variables between two groups was evaluated using Wilcoxon rank sum statistic. Differences in continuous variables across multiple groups were evaluated using the Kruskal-Wallis test. For differences in categorical variables, either exact Fisher test or Cochran–Mantel–Haenszel test was used depending on whether there were two groups or more. Changes from baseline in body weight and BMI (kg/m^2^) were assessed by duration of program participation (≤ 6 months, 7–12 months, 13–24 months) for the overall sample and after stratifying by diet type, diabetes status, hypertension status and BMI category (<30; ≥30<35; ≥35<40; ≥40). Correlations between changes in other biometric parameters (BP, FBG, TG, TC/HDL) and change in weight were also examined. Changes in self-reported medication use and the relationship with weight loss were investigated. Changes in medication were self-reported based upon ‘yes/no’ responses in the HRA (‘Are you taking any of the following medications: cholesterol medication, BP medication, oral diabetes medication, insulin for diabetes’). We also explored changes in PA (as measured by changes in estimated PA calories) and diet and the association with weight loss.

A Markov simulation model was used to project potential avoidance of diabetes and cardiovascular disease (CVD) events, specifically stroke, myocardial infarction and congestive heart failure, as a result of weight loss through participation in HMR clinic-based program in the study cohort. The distribution among CVD events was based on American Heart Association disease and stroke statistics [14]. Diabetes and CVD events avoided and associated cost savings were projected for a 5-year time horizon. Framingham risk equations were used to link weight/BMI and diabetes and CVD events [15,16]. The adjusted variables included in the risk equation are age, gender, systolic blood pressure, HDL cholesterol, triglyceride level, fasting glucose level, smoking status, parental diabetes history, and diabetes at baseline. Cost of managing diabetes and CVD events were obtained from published literature [17,18]. We applied the gender-specific weight/BMI loss efficacy of the study cohort and assumed that weight loss was maintained over 5 years. Model assumed no deaths over 5 years. Information on smoking status and parental diabetes history was not available from HRA; smoking rate was assumed using CDC report [19] and no parental diabetes history was assumed for the study cohort.

## Results

Five hundred participants were studied with duration of participation ranging from ≤ 6 to 24 months. Baseline characteristics of the participants are shown in Table 1. Overall, 67% were female, mean age 54.1 years, mean weight 243.5 lbs (range 144.0–545.0), and mean BMI 38.8 kg/m^2^ (range 25.4–85.0), including 37% with severe obesity of BMI ≥ 40. Comorbid conditions common among these patients were: diabetes or pre-diabetes (50%), high or moderate risk for dyslipidemia (60%), and hypertension or pre-hypertension (86%). Those who had longer duration of participation had higher baseline weight and BMI and tended to have a greater proportion of severe obesity and diabetes. (Table 1)

**Table 1:** Patient baseline characteristics by duration of program participation.

	Overall	Duration of Participation (months)			p-value
		≤ 6	7–12	13–24	
N (%)	500 (100)	165 (33.0)	140 (28.0)	195 (39.0)	
Female, (%)	335 (67.0)	122 (73.9)	87 (62.1)	126 (64.6)	0.06
Age, years	54.1 (11.6)	52.8(12.4)	53.6(11.5)	55.5(11.0)	0.05
Weight, pounds^1^	243.5 (62.1)	232.0(56.6)	250.1(69.6)	248.5(59.8)	0.01
BMI, kg/m^2^	38.8 (8.4)	37.7(8.2)	39.0(9.2)	39.6(7.8)	0.02
Total Cholesterol, mg/dL^2^	186.2 (38.0)	189.8 (38.7)	187.5(36.4)	182.1(38.3)	0.24
LDL-C, mg/dL^2^	106.7 (32.5)	107.8 (31.4)	108.1(31.5)	104.8(34.1)	0.55
HDL-C, mg/dL^2^	50.5 (15.0)	53.7(16.9)	50.2(13.6)	48.1(13.9)	0.00
Triglyceride, mg/dL^3^	146.3 (83.0)	140.3 (79.7)	150.2(84.3)	148.5(84.8)	0.48
TC/HDL Ratio	3.9 (1.1)	3.6 (1.1)	3.9 (1.1)	4.0 (1.1)	0.04
FBG, mg/dL ^4^	107.1 (33.6)	104.9 (30.4)	107.9 (31.1)	108.5 (37.7)	0.29
Severity of Obesity, %					0.12
Overweight	56 (11.2)	21 (12.7)	17 (12.1)	18 (9.2)	
Class I	127 (25.4)	50 (30.3)	35 (25.0)	42 (21.5)	
Class II	131 (26.2)	44 (26.7)	40 (28.6)	47 (24.1)	
Severe	186 (37.2)	50 (30.3)	48 (34.3)	88 (45.1)	
Diabetes, %					0.20
No Diabetes	251 (50.2)	91 (55.1)	67 (47.9)	93 (47.7)	
Pre-Diabetes	129 (25.8)	40 (24.2)	43 (30.7)	46 (23.6)	
Diabetes	120 (24.0)	34 (20.6)	30 (21.4)	56 (28.7)	
Dyslipidemia risk, %					0.66
Low risk	208 (41.8)	70 (42.7)	54 (38.9)	84 (43.3)	
Moderate risk	77 (15.5)	29 (17.7)	19 (13.7)	29 (14.9)	
High risk	212 (42.7)	65 (39.6)	66 (47.5)	81 (41.8)	
Hypertension, %					0.51
No Hypertension	72 (14.4)	21 (12.7)	22 (15.7)	29 (14.9)	
Pre-Hypertension	169 (33.8)	64 (38.8)	41 (29.3)	64 (32.8)	
Hypertension	259 (51.8)	80 (48.5)	77 (55.0)	102 (52.3)	

^1^ kilogram = 0.45*pound+

^2^Cholesterol mmol/L = mg/dL*0.0258

^3^Triglycerides mmol/L = mg/dl*0.0113

^4^Fasting Blood Glucose mmol/L = mg/dl*0.0555

Weight loss efficacy overall, by type of HMR diet, and by duration of program participation is shown in Table 2. Patients achieved substantial weight loss regardless of duration of participation or diet option. Overall, patients on average lost 47.7 pounds (18.9%) of IBW. The mean percent of IBW loss was 16.4%, 19.3%, 20.7% for ≤6 months (n = 165), 7–12 months (n = 140), 13–24 months (n = 195) of program participation, respectively. Substantial reduction in IBW was achieved by 6 months of program participation for both Decision Free and Healthy Solutions diet options. However, patients participating in the Decision Free program tended to have higher baseline weight/BMI and greater percent of weight loss. For both programs, longer duration of participation was associated with greater percent weight loss (Table 2).

**Table 2:** Weight loss effectiveness overall, by type of program, and by duration of program participation.

	Overall	Duration of Participation (months)			p Value
		≤ 6	7–12	13–24	
**Overall**					
N, %	500	165 (33.0)	140 (28.0)	195 (39.0)	.
Baseline Weight	243.5 (62.1)	232.0 (56.6)	250.1 (69.6)	248.5 (59.8)	0.01
Baseline BMI	38.8 (8.4)	37.7 (8.2)	39.0 (9.2)	39.6 (7.8)	0.02
Change in Weight	-47.7 (31.3)	-38.5 (18.9)	-49.6 (29.2)	-54.1 (38.5)	0.00
Change in BMI	-7.7 (5.1)	-6.4 (3.6)	-7.8 (4.4)	-8.6 (6.2)	0.00
% Change in Weight	-18.9 (9.5)	-16.4 (6.2)	-19.3 (8.6)	-20.7 (11.7)	0.00
**Decision Free**					
N, %	351	103 (29.3)	102 (29.1)	146 (41.6)	.
Baseline Weight	249.0 (61.6)	240.9 (59.4)	251.7 (72.0)	252.8 (54.6)	0.12
Baseline BMI	39.7 (8.5)	39.1 (8.6)	39.3 (9.8)	40.3 (7.3)	0.07
Change in Weight	-51.0 (32.9)	-41.7 (20.7)	-52.6 (30.7)	-56.4 (39.7)	0.00
Change in BMI	-8.3 (5.4)	-7.0 (4.1)	-8.3 (4.6)	-9.2 (6.4)	0.01
% Change in Weight	-19.8 (9.9)	-17.2 (6.7)	-20.3 (8.5)	-21.3 (12.1)	0.00
**Healthy Solutions**					
N, %	149	62 (41.6)	38 (25.5)	49 (32.9)	.
Baseline Weight	230.6 (61.7)	217.2 (48.4)	245.7 (63.3)	235.8 (72.3)	0.06
Baseline BMI	36.8 (7.7)	35.5 (6.8)	38.1 (7.4)	37.4 (8.9)	0.23
Change in Weight	-39.9 (25.3)	-33.2 (14.2)	-41.6 (23.4)	-47.3 (34.4)	0.07
Change in BMI	-6.2 (3.8)	-5.4 (2.3)	-6.5 (3.5)	-7.1 (5.3)	0.18
% Change in Weight	-16.8 (8.0)	-15.2 (5.2)	-16.6 (8.1)	-19.0 (10.2)	0.13

kilogram = 0.45*pound+

Weight loss efficacy was examined by baseline risk level including severity of obesity, status of diabetes, hypertension, and dyslipidemia risk. Weight loss efficacy was most pronounced in those who had severe obesity (BMI ≥40) at baseline. In our cohort of participants, nearly 40% had severe obesity; the average weight loss in these patients was 22% compared to 19%, 17%, and 14% for class II obesity, class I obesity, and overweight patients, respectively (Table 3). This observation could be due to the higher IBW of severely obese patients as well as a higher proportion of high BMI patients having longer program participation of 13–24 months as shown in Table 1.

**Table 3:** Weight loss efficacy (mean, sd) by severity of obesity.

	Severe Obesity BMI ≥ 40	Class II obesity 35 ≤ BMI < 40	Class I obesity 30 ≤ BMI < 35	Overweight BMI < 30
N (%)	186 (37.2)	131 (26.2)	127 (25.4)	56 (11.2)
Baseline Weight	299.3 (58.7)	234.3 (29.2)	201.7 (23.7)	174.5 (22.3)
Baseline BMI	47.45 (6.9)	37.2 (1.4)	32.5 (1.5)	28.2 (1.3)
Change in Weight	-65.5 (37.9)	-45.4 (21.5)	-34.1 (17.3)	-25.0 (15.1)
Change in BMI	-10.7 (5.9)	-7.3 (3.7)	-5.5 (3.0)	-3.6 (2.3)
% Change Weight	-21.6 (10.8)	-19.2 (8.4)	-16.8 (8.2)	-14.1 (6.9)

kilogram = 0.45*pound+

Weight loss efficacy in patients with high risk comorbidity, such as diabetes, pre-diabetes, hypertension, and dyslipidemia were also examined. Patients with diabetes and hypertension tended to achieve slightly greater % change in IBW loss compared to those without (19.5% vs. 18.7% and 19.4% vs. 17.5%, respectively). All patient subgroups showed relatively consistent weight loss efficacy ranging around 17–20%.

Improvement in other biometric parameters was also observed for BP, FBG, and lipid profile (Table 4). Overall, mean (standard deviation) improvement was 6.6 (16.5) mmHg in systolic BP, 4.2 (12.0) mmHg in diastolic BP, 10.9 (27.9) mg/dL in FBG, 36.0 (77.0) mg/dL in triglyceride, and 0.4 (0.9) in total cholesterol to HDL ratio. Improvement in the lipid profile appeared to be greater with longer program participation whereas systolic BP and blood glucose improvements were observed with program participation of ≤6 months or longer.

**Table 4:** Changes from baseline in biometric parameters by duration of program participation.

	Overall	Duration of Participation (months)			p value
		≤ 6	7–12	13–24	
N, %	500	165 (33.0)	140 (28.0)	195 (39.0)	.
**Blood Pressure (BP), mmHg**					
Baseline Systolic BP	126.7 (14.9)	126.8 (14.1)	126. (15.9)	126.8 (14.7)	0.81
Change in Systolic BP	-6.6 (16.5)	-7.6 (15.8)	-6.4 (17.1)	-5.8 (16.8)	0.27
% Change in Systolic BP	-4.2 (12.7)	-5.1 (12.6)	-4.2 (12.9)	-3.7 (12.6)	0.27
Baseline Diastolic BP	78.1 (10.9)	78.6 (11.4)	78.0 (9.0)	77.8 (11.6)	0.27
Change in Diastolic BP	-4.2 (12.0)	-4.7 (12.2)	-3.9 (10.6)	-4.0 (12.9)	0.49
% Change in Diastolic BP	-3.4 (28.6)	-2.1 (45.6)	-4.2 (13.4)	-3.9 (14.5)	0.51
**Fasting Blood Glucose (FBG), mg/dL**^1^					
Baseline FBG	107.1 (33.6)	104.9 (30.4)	107.9 (31.1)	108.5 (37.7)	0.29
Change in FBG	-10.9 (27.9)	-10.9 (22.9)	-12.2 (32.7)	-10.0 (28.1)	0.19
% Change in FBG	-7.1 (19.4)	-7.8 (14.4)	-7.9 (21.2)	-5.9 (21.7)	0.15
**Triglycerides, mg/dL**^2^					
Baseline Triglycerides	146.3 (83.0)	140.3 (79.7)	150.2 (84.3)	148.5 (84.8)	0.48
Change in Triglycerides	-36.0 (77.0)	-23.8 (70.9)	-44.1 (79.7)	-40.6 (79.0)	0.00
% Change in Triglycerides	-16.2 (38.1)	-8.3 (38.7)	-20.6 (37.2)	-19.8 (37.4)	0.00
**TC/HDL Ratio**					
Baseline TC/HDL Ratio	3.9 (1.1)	3.7 (1.1)	3.9 (1.1)	4.00 (1.1)	0.04
Change in TC/HDL Ratio	-0.4 (0.9)	-0.1 (0.9)	-0.5 (0.9)	-0.5 (1.0)	0.00
% Change in TC/HDL Ratio	-7.3 (21.6)	-0.6 (22.0)	-10.7 (20.0)	-10.6 (21.2)	0.00

^1^Fasting Blood Glucose mmol/L = mg/dl*0.0555;

^2.^Triglycerides mmol/L = mg/dl*0.0113

There is a significant correlation (p< 0.01) between change in weight and change in diastolic BP (correlation coefficient ρ = 0.188), systolic blood pressure (ρ = 0.110), FBG (ρ = 0.176), and total cholesterol/ HDL ratio (ρ = 0. 295). Observation of changes in these biometric parameters did not take into consideration the impact of weight loss on self-reported medication use due to potential improvement in health status.

Among 192 (38%), 235 (46%), and 67 (15%) patients who reported taking oral anti-dyslipidemia, anti-hypertensive, and anti-diabetic medications at baseline, 43 (22%), 79 (33%), and 27 (40%) reported no longer taking the medication at follow up. Compared to those who continued to take medication, greater weight loss was observed in those who reported having discontinued medication: 25% vs. 17% in those stopped vs continuing anti-dyslipidemia; 22% vs. 17% in those who stopped vs. continuing anti-hypertensive; 23% vs. 18% in those who stopped vs. continuing oral anti-diabetic medication (Table 5).

**Table 5:** Weight loss efficacy (mean, sd) by status of medication use.

	No Medication at baseline		Medication at baseline		
	No medication at follow-up	Medication at follow-up	No medication at follow-up	Medication at follow-up	p-value
**Cholesterol medication**					
N (%)	295 (59.0)	13 (2.6)	43 (8.6)	149 (29.8)	
Baseline Weight	237.7 (57.9)	271.2 (75.7)	251.7 (56.40)	250.2 (69.1)	0.08
Change Weight	-46.3 (28.6)	-55.69 (34.2)	-64.9 (38.6)	-44.8 (32.4)	0.00
% Change Weight	-18.9 (9.1)	-19.67 (10.6)	-24.74 (10.7)	-17.18 (9.2)	0.00
**Anti-hypertensive**					
N (%)	253 (50.6)	12 (2.40)	79 (15.8)	156 (31.2)	
Baseline Weight	228.6 (50.8)	257.2 (60.3)	248.9 (51.1)	264.0 (76.4)	0.00
Change Weight	-44.0 (27.2)	-53.2 (30.2)	-57.9 (35.9)	-48.1 (33.9)	0.00
% Change Weight	-18.6 (9.2)	-20.1 (8.5)	-22.4 (10.7)	-17.4 (8.8)	0.00
**Oral Anti-diabetics**					
N, %	391 (85.4)	-	27 (5.90)	40 (8.73)	
Baseline Weight	236.4 (56.3)	-	272.3 (57.6)	253.0 (83.6)	0.00
Change Weight	-45.7 (27.6)	-	-63.0 (41.1)	-46.4 (30.4)	0.08
% Change Weight	-18.8 (9.0)	-	-22.2 (12.5)	-17.5 (6.7)	0.35

Kilogram = 0.45*pound+

Physical activity level was reported at both baseline and follow-up in 403 out of the 500 patients. There were no notable differences in baseline characteristics between those included (n = 403) and not included (n = 97) in the PA assessment. Patient’s self-reported PA level at follow-up increased by 1,403 calories per week on average from baseline. Increase in PA level appeared to be significantly correlated with weight loss (ρ = -0.22; p < 0.0001) but not with other biometric parameters.

Dietary behavior was reported as consumption of low or high/moderate fat diet and V/F consumption per week. At baseline, only 22% of patients reported eating a low fat diet compared to 94% at follow-up. Similarly, only 28% of patients reported consuming V/F ≥6–7 days per week vs. 87% eating that amount at follow-up. Improvement in dietary behaviors, such as shift from high fat diet to low fat diet, appeared to be associated with slightly more weight loss compared to maintaining good dietary behaviors (51.2 lbs. vs. 40.1 lbs. (p = 0.00), respectively).

Using a simulation model, it was estimated that, among the study cohort of 500 patients, weight loss achieved through participation in the HMR clinic programs could result in 22 diabetes events, 30 CVD events and $1,992,370 in health care costs avoided over 5 years. Among 120 patients with diabetes, 22 CVD events and $1,022,196 could be avoided over 5 years. Among 186 patients with severe obesity (BMI ≥40), 6 diabetes events, 17 CVD events, and $958,958 could be avoided over 5 years.

## Discussion

In a large, real world cohort of overweight and obese patients who received HILI through participation in the HMR clinic-based program, we found that the patients were able to achieve clinically significant weight loss ranging between 15–17% within 6 months of program participation. There is a trend of greater weight reduction in those who had longer participation in the program for up to 24 months. Previous studies of HMR clinic-based programs from shorter duration randomized, controlled studies that were conducted in small cohorts of high risk patients reported similar findings [9,10,20].

The current study highlights the weight loss efficacy that may be achieved by patients who participate in a clinic-based weight management program regardless of duration of participation, risk profile, and comorbidity status. The findings from this study demonstrate that all patients were able to achieve weight loss of > 15% relative to their IBW, with greater weight loss efficacy associated with longer duration of participation. Similarly, individuals with the highest BMI (i.e., ≥ 40 kg/m^2^) were observed to achieve the greatest weight loss efficacy, as assessed by % change in body weight; however, this observation may be confounded by a longer duration of participation by these individuals. This observation is an important one given that those patients with the highest baseline weight had a greater tendency to have co-morbid conditions of dyslipidemia, hypertension and diabetes and were more likely to be taking medications for these conditions. The impact of weight loss on medication utilization cannot be understated, with 22.4%, 38.7% and 40.3% of individuals reporting discontinued use of medications for treatment of dyslipidemia, hypertension and diabetes, respectively. Although discontinuation of medications may not have the greatest impact to the overall health care costs for these individuals, the elimination of medications may be associated with reduction in potential side effects and improvement in quality of life. Our observations are in keeping with the Look AHEAD and other studies [20,21], which have also demonstrated that participants in HILI had greater reductions in medication use and cost, particularly for diabetes medications, compared to those in less intensive usual care treatments.

Although the health benefits achieved by patients in this study appear to be primarily mediated by weight loss, it is possible that lifestyle behaviors acquired and reinforced in the clinic-based program may have contributed to these effects independently. For example, patients in this study demonstrated a 1400 kcal/week increase in PA relative to baseline. The association of PA with improved insulin sensitivity and reduced utilization of diabetes medications has been well documented [22,23], which may have explained, in part, the reductions in use of diabetes medications that were reported by some individuals in this study. As well, over 90% of individuals were observed to have a low-fat diet score at follow up, and nearly 90% had increased their F/V intake. Although the relationship between lifestyle changes and biometric changes in this study were not statistically significant, it is possible that the duration of the study was not sufficient to detect an independent effect beyond weight loss itself.

The behavioral based program delivered in this study is consistent with obesity treatment guidelines that have been issued by a number of bodies [7,8], which specify that HILI is fundamental to ensuring that patients achieve clinically significant weight loss. Such interventions include high frequency contact (e.g., ≥ 14 group or individual sessions in the first 6 months), behavioral interventions with trained interventionists, personalized feedback, and continued, albeit less frequent contact (i.e., at least once per month), during weight maintenance. More intensive lifestyle intervention such as delivered in this study (i.e., weekly coaching provided for the duration of weight loss and weight maintenance phases, which enables lifestyle skill acquisition and practice with individualized patient goals based upon data collection) may have, in part, contributed to enhanced weight loss efficacy. Obesity treatment guidelines also recommend a weight loss goal of 5–10% of IBW in the first 6 months for patients with obesity. Patients who participated for ≤ 6 months in this study were observed to have a mean change in weight of 16.4%, which greatly exceeds treatment guidelines. Furthermore, literature has shown that early weight loss results are strong motivator for long-term behavior change [24].

While cost of participation in programs that maintain structured diets through the use of PCFs, such as HMR, may be perceived as a barrier to some individuals, it is important to note that use of PCF is one of the main drivers to achieving the desired weight loss by effectively managing caloric intake. The cost of these foods would replace largely what an individual is already spending for food, including grocery items and eating outside the home. According to a consumer research study of a 7-day online diary conducted among 502 obese individuals, the average daily amount spent on food was approximately $13.60. This is roughly the daily cost of the HMR PCF. Given that many individuals are eating outside the home more frequently, it is not uncommon for overall food expenses to decrease with participation in a clinic-based program that utilizes PCFs. This is also an important point to consider when comparing commercial weight-loss programs, many of which do not take into consideration that all participants are spending money on food, regardless of the program that they are participating in.

Viable and durable management options for individuals with obesity are imperative in light of the increasing prevalence of obesity and its associated co-morbid conditions such as diabetes and cardiovascular disease. The medical expenditures associated with the care of these individuals are not sustainable when considering the impact on the gross domestic product. Annual medical costs have been estimated based upon starting BMI and % change in BMI for individuals with and without diabetes. [25] Annual medical costs were estimated to increase by over $3500 for US adults with obesity, with greater medical expenditures projected for BMI ≥ 35 and for individuals with diabetes. In another study that examined claims data for employees who participated in annual health assessment, researchers found that the average annual health care costs for a person with a BMI of 45 were over 2-fold higher relative to a person with a BMI of 19 [26]. The direct costs of obesity cannot be denied and, importantly, are likely to under-represent the total cost impact when one considers the indirect costs that may also be associated with obesity, especially in the workplace (e.g., presenteeism, absenteeism, reduced productivity, etc.).

Weight loss through intensive lifestyle intervention, such as provided in this study, could be an economic approach to preventing and managing diabetes and cardiovascular diseases, two chronic diseases associated with substantial clinical and financial burden to payers, employers, and health care systems, particularly in high risk patients such as those with diabetes or severe obesity. For example, we projected 5-year cost savings of $1,022,196 in 120 patients with diabetes due to CVD events prevented based upon the weight loss efficacy achieved in this study. Assuming all patients with diabetes in our study cohort participated in the program for 24 months, it would have resulted in a cost-saving of approximately $350,000 in 5 years.

The estimated out-of-pocket costs for patients with diabetes participating in the 24-month clinic-based HMR program (6-month weight loss phase and 18-month weight maintenance phase) with medical supervision is approximately $5,600 per person, including costs of in-person coaching, phone support, medical monitoring, laboratory testing, and PCF. It is important to note, however, that over half of the estimated costs of the initial 6 months are related to PCFs, which, in essence, replace the foods that individuals are consuming prior to their participation in the program (e.g., grocery shopping, eating outside the home). Taken together, the actual out-of-pocket cost of the full program is considerably less (e.g., ~$3400) given that the cost of PCFs is not an added expense for most individuals as food must be purchased regardless of participation in the HMR program. Additionally, for some patients, some of the medical and lab fees are reimbursable from health insurance. It is acknowledged that a more complete evaluation of the economic value would require the full cost of the HMR program, not just costs from the patient’s perspective.

The data from this study further underscore the importance enabling patient access to the full continuum of care of evidence-based obesity preventive and treatment modalities, with HILI and behavioral treatments serving as the foundation and complemented by other adjunct interventions such as pharmacotherapy and/or bariatric surgery/device, when appropriate. This also speaks to the need for the development of quality measures that facilitate the treatment of these patients for the optimization of population health. While the importance of healthcare quality measurement to improve outcomes is clear, there are very few quality measures established to drive optimal outcomes for people with obesity.

Medicare Access and Children’s Health Insurance Program (CHIP) Reauthorization Act of 2015 will take into account how clinical best practices and clinical practice guidelines should be used in the development of quality measures. Presently, no useful quality measure currently exists for what should happen after a patient is diagnosed with obesity. In most practices, a patient may be diagnosed, the screening measure checked as completed, and no further steps taken, outcomes are not recorded, or may be limited to only reporting weight. This is most certainly subject to change as more health systems gravitate toward emerging value-based models and as Medicare providers could conceivably earn payment adjustment based on evidence-based and practice-specific quality data that are currently relevant to treating individuals with obesity. Such shifts and evidence-based weight management programs, such as HMR, will greatly enhance provider ability to focus on keeping people healthy long-term, rather than treating them only when sick.

It is acknowledged that this study has limitations. Firstly, the retrospective design of the study limited the ability to determine patient retention following enrollment and to determine the outcomes for those individuals who did not remain in the program, which is likely to result in an overestimation of the program benefits. According to internal HMR data, the retention rate at 6 months is approximately 50%, on average. It is important to note that these are real world data where patients voluntarily enroll and pay for the treatment. The retention rate at HMR compares favorably to real world data published by other treatment programs, such as 26% reported from Medifast [27] and 22% reported from Jenny Craig [28] According to internal HMR data, approximately 37% of patients are retained at one year which is more than three times higher than 10.4% reported in Diabetes Prevention Recognition Programs. Most programs for the treatment of obesity do not keep or report such data and randomized controlled studies are not comparable given that most offer retention incentives that are not available outside of the study. Secondly, the data in this study are limited to HMR clinic-based participants, and it is widely acknowledged that weight management solutions are not “one size fits all”. Furthermore, not all individuals who are seeking treatment live or work in close proximity to a clinic-based program, which highlights the need for a spectrum of solutions, including less structured “do-it-yourself” options and remotely-delivered programs in which the weekly group coaching intervention is delivered by telephone. Although the HMR self-directed and remote programs are associated with less robust weight loss efficacy compared to the clinic-based program, these more flexible options are still associated with clinically meaningful weight loss [29,30]. Lastly, we projected clinical and economic impact of weight reduction focusing only on potential diabetes and CVD events avoided. It was not a thorough economic assessment and was likely an under-estimate of the holistic benefit of weight loss. It was also not aimed to take into consideration cost-effectiveness of the HMR clinic-based program which would involve considerations such as the cost of program delivery (e.g., employee salaries, clinic space considerations, etc.), the avoidance of other obesity-related conditions and/or treatments (e.g., pharmacotherapy or bariatric surgery), and impact on quality of life. A more complete evaluation of the economic value would require the inclusion of full cost of HMR program delivery as well as the costs of weight maintenance beyond the 2-year program.

In summary, in this real-world study of a large cohort of obese patients, our results show encouraging success in the implementation of a clinic-based HILI program which resulted in weight loss efficacy that surpasses the weight loss goals as outlined in obesity treatment guidelines. The weight loss achieved in this study was associated with reduction in self-reported medication utilization and projected avoidance of incident cases of diabetes and CVD events. The potential to prevent diabetes is not surprising when one considers that the mean weight loss efficacy achieved by patients in this study exceeded the CDC Diabetes Prevention Recognition Program weight loss benchmark (i.e., 5–7%) by approximately 3-fold. Taken together, clinic-based programs such as those provided by HMR are a viable option for health care organizations seeking to optimize the delivery of care in their patient population who are overweight or obese to avoid costly chronic diseases through a life-long healthy life style.

## Supporting information

S1 File HILI and clinical outcomes_data file.xlsx. (XLS)

S2 File HMR data dictionary.xlsx. (XLSX)

S1 Table Patient baseline characteristics by duration of program participation: Standard international unit metric system with 95% confidence interval. (PDF)

S2 Table Weight loss effectiveness overall, by type of program, and by duration of program participation: Standard international. (PDF)

S3 Table Weight loss efficacy (mean, sd) by severity of obesity: Standard international unit metric system with 95% confidence interval. (PDF)

S4 Table Changes from baseline in biometric parameters by duration of program participation: Standard international unit metric system with 95% confidence interval. (PDF)

S5 Table Weight loss efficacy (mean, sd) by status of medication use: Standard international unit metric system with 95% confidence interval. (PDF)
