# Supplementary material for: High intensity lifestyle intervention and long-term impact on weight and clinical outcomes
Source: PLoS One. 2018 Apr 18;13(4):e0195794. doi: 10.1371/journal.pone.0195794 (PMC5905976; doi:10.1371/journal.pone.0195794)
Supplement: S1 Table — (PDF) [file pone.0195794.s003.pdf]

Supporting Information

S1 Table: Patient Baseline Characteristics by Duration of Program Participation: Standard International Unit Metric System with 95% confidence interval

	Overall	Duration of Participation (months)			p-value
		≤ 6	7-12	13-24	
N (%)	500 (100)	165 (33.0)	140 (28.0)	195 (39.0)	
Female, N (%)	335 (67.0)	122 (73.9)	87 (62.1)	126 (64.6)	0.06
Age, years, mean (SD)	54.1 (11.6)	52.8(12.4)	53.6(11.5)	55.5(11.0)	0.05
(95%CI)	(53.1, 55.1)	(50.1, 54.7)	(51.7, 55.5)	(54.0, 57.1)	
Weight Kg, Mean (SD)	110.5 (28.2)	105.2 (25.7)	113.4 (31.6)	112.7 (27.1)	0.01
(95% CI)	(108.0, 112.9)	(101.3, 109.2)	(108.2, 118.7)	(108.9, 116.6)	
BMI, kg/m^2, mean (SD)	38.8 (8.4)	37.7(8.2)	39.0(9.2)	39.6(7.8)	0.02
(95% CI)	(38.1, 39.5)	(36.5, 39.0)	(37.5, 40.5)	(38.5, 40.7)	
Total Cholesterol mmol/L, Mean (SD)	4.8 (1.0)	4.9 (1.0)	4.9 (0.9)	4.7 (1.0)	0.24
(95% CI)	(4.7, 4.9)	(4.8, 5.1)	(4.7, 5.0)	(4.6, 4.9)	
LDL-C mmol/L, Mean (SD)	2.8 (0.8)	2.8 (0.8)	2.8 (0.8)	2.7 (0.9)	0.55
(95% CI)	(2.7, 2.8)	(2.7, 2.9)	(2.7, 2.9)	(2.6, 2.8)	
HDL-C mmol/L, Mean (SD)	1.3 (0.4)	1.4 (0.4)	1.3 (0.4)	1.2 (0.4)	0.00
(95% CI)	(1.3, 1.3)	(1.3, 1.5)	(1.2, 1.4)	(1.2, 1.3)	
Triglycerides mmol/L, Mean (SD)	1.7 (0.9)	1.6 (0.9)	1.7 (1.0)	1.7 (1.0)	0.48
(95% CI)	(1.6, 1.7)	(1.4, 1.7)	(1.5, 1.9)	(1.5, 1.8)	
Cholesterol/HDL Ratio, Mean (SD)	3.9 (1.1)	3.7 (1.1)	3.9 (1.1)	4.0 (1.1)	0.04
(95% CI)	(3.8, 4.0)	(3.6, 3.9)	(3.8, 4.1)	(3.8, 4.2)	
FBG mmol/L, Mean (STD)	5.9 (1.9)	5.8 (1.7)	6.0 (1.7)	6.0 (2.1)	0.29
(95% CI)	(5.8, 6.1)	(5.6, 6.1)	(5.7, 6.3)	(5.7, 6.3)	
Severity of Obesity, %					0.12
Overweight	56 (11.2)	21 (12.7)	17 (12.1)	18 (9.2)	
Class I	127 (25.4)	50 (30.3)	35 (25.0)	42 (21.5)	
Class II	131 (26.2)	44 (26.7)	40 (28.6)	47 (24.1)	
Severe	186 (37.2)	50 (30.3)	48 (34.3)	88 (45.1)	
Diabetes, %					0.20
No Diabetes	251 (50.2)	91 (55.1)	67 (47.9)	93 (47.7)	
Pre-Diabetes	129 (25.8)	40 (24.2)	43 (30.7)	46 (23.6)	
Diabetes	120 (24.0)	34 (20.6)	30 (21.4)	56 (28.7)	

Dyslipidemia risk, %					0.66
Low risk	208 (41.8)	70 (42.7)	54 (38.9)	84 (43.3)	
Moderate risk	77 (15.5)	29 (17.7)	19 (13.7)	29 (14.9)	
High risk	212 (42.7)	65 (39.6)	66 (47.5)	81 (41.8)	
Hypertension, %					0.51
No Hypertension	72 (14.4)	21 (12.7)	22 (15.7)	29 (14.9)	
Pre-Hypertension	169 (33.8)	64 (38.8)	41 (29.3)	64 (32.8)	
Hypertension	259 (51.8)	80 (48.5)	77 (55.0)	102 (52.3)	
