# Supplementary material for: High intensity lifestyle intervention and long-term impact on weight and clinical outcomes
Source: PLoS One. 2018 Apr 18;13(4):e0195794. doi: 10.1371/journal.pone.0195794 (PMC5905976; doi:10.1371/journal.pone.0195794)
Supplement: S2 Table — (PDF) [file pone.0195794.s004.pdf]

Supporting Information

S2 Table: Weight Loss Effectiveness Overall, By Type of Program, and By Duration of Program Participation: Standard International Unit Metric System with 95% confidence interval

	Overall	Duration of Participation (months)			p Value
		≤ 6	7-12	13-24	
**Overall**					
N, %	500	165 (33.0)	140 (28.0)	195 (39.0)	
Baseline Weight Kg, Mean(SD)	110.5 (28.2)	105.2 (25.7)	113.4 (31.6)	112.7 (27.1)	0.01
(95% CI)	(108.0, 112.9)	(101.3, 109.2)	(108.2, 118.7)	(108.9, 116.6)	
Baseline BMI, kg/m^2 Mean (SD)	38.8 (8.4)	37.7 (8.2)	39.0 (9.2)	39.6 (7.8)	0.02
(95% CI)	(38.1, 39.5)	(36.5, 39.0)	(37.5, 40.5)	(38.5, 40.7)	
Change Weight Kg, Mean (SD)	-21.6 (14.2)	-17.5 (8.6)	-22.5 (13.2)	-24.5 (17.5)	0.00
(95% CI)	(-22.9, -20.4)	(-18.8, -16.1)	(-24.7, -20.3)	(-27.0, -22.1)	
Change in BMI, Mean (SD)	-7.7 (5.1)	-6.4 (3.6)	-7.8 (4.4)	-8.6 (6.2)	0.00
(95% CI)	(-8.1, -7.2)	(-7.0, -5.8)	(-8.5, -7.1)	(-9.5, -7.7)	
% Change Weight, Mean (SD)	-18.9 (9.5)	-16.4 (6.2)	-19.3 (8.5)	-20.7 (11.7)	0.00
(95% CI)	(-19.7, -18.1)	(-17.4, -15.5)	(-20.7, -17.9)	(-22.4, -19.1)	
% Change BMI, Mean (SD)	-19.1 (10.4)	-16.7 (7.2)	-19.5 (9.0)	-20.8 (13.0)	0.00
(95%)	(-20.0, -18.2)	(-17.8, -15.6)	(-21.0, -18.0)	(-22.6, -19.0)	
**Decision Free**					
N, %	351	103 (29.3)	102 (29.1)	146 (41.6)	
Baseline Weight Kg, Mean(SD)	112.9 (27.9)	109.3 (27.0)	114.2 (32.7)	114.7 (24.8)	0.12
(95% CI)	(110.0, 115.9)	(104.0, 114.6)	(107.8, 120.6)	(110.6, 118.7)	
Baseline BMI, Mean (SD)	39.7 (8.5)	39.1 (8.6)	39.3 (9.8)	40.3 (7.3)	0.07
(95% CI)	(38.8, 40.5)	(37.4, 40.8)	(37.4, 41.2)	(39.1, 41.5)	
Change Weight Kg, Mean (SD)	-23.1 (14.9)	-18.9 (9.4)	-23.9 (13.9)	-25.6 (18.0)	0.00
(95% CI)	(-24.7, -21.6)	(-20.8, -17.1)	(-26.6, -21.1)	(-28.5, -22.6)	

Change in BMI, Mean (SD)	-8.3 (5.4)	-7.0 (4.1)	-8.3 (4.6)	-9.1 (6.4)	0.01
(95% CI)	(-8.8, -7.7)	(-7.8, -6.2)	(-9.2, -7.4)	(-10.2, -8.1)	
% Change Weight, Mean (SD)	-19.8 (9.9)	-17.2 (6.7)	-20.3 (8.5)	-21.3 (12.1)	0.00
(95% CI)	(-20.8, -18.8)	(-18.5, -15.9)	(-22.0, -18.6)	(-23.3, -19.3)	
% Change in BMI, Mean (SD)	-20.2 (10.9)	-17.6 (7.9)	-20.5 (9.1)	-21.8 (13.2)	0.00
(95% CI)	(-21.3, -19.1)	(-19.1, -16.0)	(-22.3, -18.7)	(-24.0, -19.7)	
**Healthy Solutions**					
N, %	149	62 (41.6)	38 (25.5)	49 (32.9)	
Baseline Weight Kg, Mean(SD)	104.6 (28.0)	98.5 (21.9)	111.5 (28.7)	107.0 (32.8)	0.06
(95% CI)	(100.1, 109.1)	(92.9, 104.1)	(102.0, 120.9)	(97.5, 116.4)	
Baseline BMI, Mean (SD)	36.8 (7.7)	35.5 (6.8)	38.1 (7.4)	37.4 (8.9)	0.23
(95% CI)	(35.5, 38.0)	(33.7, 37.2)	(35.7, 40.6)	(34.9, 40.0)	
Change Weight Kg, Mean (SD)	-18.1 (11.5)	-15.0 (6.5)	-18.9 (10.6)	-21.4 (15.6)	0.07
(95% CI)	(-20.0, -16.3)	(-16.7, -13.4)	(-22.4, -15.4)	(-25.9, -17.0)	
Change BMI, Mean (SD)	-6.2 (3.9)	-5.4 (2.3)	-6.5 (3.5)	-7.1 (5.3)	0.18
(95% CI)	(-6.9, -5.6)	(-6.0, -4.8)	(-7.7, -5.4)	(-8.6, -5.5)	
% Change Weight, Mean (SD)	-16.8 (8.0)	-15.2 (5.2)	-16.6 (8.1)	-19.0 (10.2)	0.13
(95% CI)	(-18.1, -15.5)	(-16.5, -13.8)	(-19.3, -14.0)	(-21.9, -16.1)	
% Change in BMI, Mean (SD)	-16.4 (8.7)	-15.2 (5.5)	-16.8 (8.2)	-17.7 (11.8)	0.31
(95% CI)	(-17.8, -15.0)	(-16.6, -13.8)	(-19.5, -14.1)	(-21.1, -14.4)	
