# Supplementary material for: High intensity lifestyle intervention and long-term impact on weight and clinical outcomes
Source: PLoS One. 2018 Apr 18;13(4):e0195794. doi: 10.1371/journal.pone.0195794 (PMC5905976; doi:10.1371/journal.pone.0195794)
Supplement: S3 Table — (PDF) [file pone.0195794.s005.pdf]

Supporting Information

S3 Table: Weight Loss Efficacy (mean, sd) by Severity of Obesity: Standard International Unit Metric System with 95% confidence interval

	Severe Obesity BMI ≥ 40	Class II obesity 35 ≤ BMI < 40	Class I obesity 30 ≤ BMI < 35	Overweight BMI < 30
N (%)	186 (37.2)	131 (26.2)	127 (25.4)	56 (11.2)
Baseline Weight, Kg	135.8 (26.6)	106.3 (13.2)	91.5 (10.7)	79.2 (10.1)
(95% CI)	(131.9, 139.6)	(104.0, 108.6)	(89.6, 93.4)	(76.5, 81.9)
Baseline BMI	47.5 (6.8)	37.2 (1.4)	32.5 (1.5)	28.2 (1.3)
(95% CI)	(46.5, 48.4)	(36.9, 37.4)	(32.2, 32.8)	(27.9, 28.6)
Change Weight Kg	-29.7 (17.2)	-20.6 (9.7)	-15.5 (7.9)	-11.3 (6.8)
(95% CI)	(-32.2, -27.2)	(-22.3, -18.9)	(-16.8, -14.1)	(-13.2, -9.5)
Change BMI	-10.7 (5.9)	-7.3 (3.7)	-5.5 (3.0)	-3.6 (2.3)
(95% CI)	(-11.5, -9.8)	(-7.9, -6.6)	(-6.0, -5.0)	(-4.2, -2.9)
% Change Weight	-21.6 (10.8)	-19.2 (8.4)	-16.8 (8.2)	-14.1 (6.9)
(95% CI)	(-23.2, -20.0)	(-20.7, -17.8)	(-18.2, -15.3)	(-16.0, -12.3)
