# Supplementary material for: High intensity lifestyle intervention and long-term impact on weight and clinical outcomes
Source: PLoS One. 2018 Apr 18;13(4):e0195794. doi: 10.1371/journal.pone.0195794 (PMC5905976; doi:10.1371/journal.pone.0195794)
Supplement: S4 Table — (PDF) [file pone.0195794.s006.pdf]

Supporting Information

S4 Table: Changes From Baseline In Biometric Parameters By Duration Of Program Participation: Standard International Unit Metric System with 95% confidence interval

	Overall	Duration of Participation (months)			p value
		≤ 6	7-12	13-24	
N, %	500	165 (33.0)	140 (28.0)	195 (39.0)	
**Blood Pressure (BP), mmHg**					
Baseline Systolic BP	126.7 (14.8)	126.8 (14.1)	126.5 (15.9)	126.8 (14.7)	0.81
(95% CI)	(125.4, 128.0)	(124.6, 128.9)	(123.8, 129.1)	(124.7, 128.8)	
Change Systolic BP	-6.6 (16.5)	-7.6 (15.8)	-6.4 (17.0)	-5.8 (16.8)	0.27
(95% CI)	(-8.0, -5.1)	(-10.0, -5.2)	(-9.2, -3.5)	(-8.2, -3.4)	
% Change Systolic BP	-4.2 (12.7)	-5.1 (12.6)	-4.1 (12.9)	-3.7 (12.6)	0.27
(95% CI)	(-5.4, -3.1)	(-7.0, -3.1)	(-6.2, -1.9)	(-5.4, -1.9)	
Baseline Diastolic BP	78.1 (10.9)	78.6 (11.4)	78.0 (9.0)	77.8 (11.6)	0.27
(95% CI)	(77.1, 79.1)	(76.8, 80.3)	(76.5, 79.5)	(76.1, 79.4)	
Change Diastolic BP	-4.2 (12.0)	-4.7 (12.2)	-3.9 (10.6)	-4.0 (12.9)	0.49
(95% CI)	(-5.2, -3.1)	(-6.6, -2.8)	(-5.7, -2.2)	(-5.8, -2.2)	
% Change Diastolic BP	-3.4 (28.6)	-2.1 (45.6)	-4.2 (13.4)	-3.9 (14.5)	0.51
(95% CI)	(-5.9, -0.9)	(-9.1, 4.9)	(-6.5, -2.0)	(-5.9, -1.8)	
**Fasting Blood Glucose (FBG), mmol/L**					
Baseline FBG	5.9 (1.9)	5.8 (1.7)	6.0 (1.7)	6.0 (2.1)	0.29
(95% CI)	(5.8, 6.1)	(5.6, 6.1)	(5.7, 6.3)	(5.7, 6.3)	
Change FBG	-0.6 (1.5)	-0.6 (1.3)	-0.7 (1.8)	-0.6 (1.6)	0.19
(95% CI)	(-0.7, -0.5)	(-0.8, -0.4)	(-1.0, -0.4)	(-0.8, -0.3)	
% Change FBG	-7.1 (19.4)	-7.8 (14.4)	-8.0 (21.2)	-5.9 (21.7)	0.15
(95% CI)	(-8.8, -5.4)	(-10.0, -5.6)	(-11.5, -4.4)	(-9.0, -2.9)	
**Triglycerides, mmol/L**					
Baseline Triglycerides	1.7 (0.9)	1.6 (0.9)	1.7 (1.0)	1.7 (1.0)	0.48

(95% CI)	(1.6, 1.7)	(1.4, 1.7)	(1.5, 1.9)	(1.5, 1.8)	
Change Triglycerides	-0.4 (0.9)	-0.3 (0.8)	-0.5 (0.9)	-0.5 (0.9)	0.00
(95% CI)	(-0.5, -0.3)	(-0.4, -0.1)	(-0.6, -0.3)	(-0.6, -0.3)	
**Total Cholesterol (TC)/HDL Ratio**					
Baseline TC/HDL Ratio	3.9 (1.1)	3.7 (1.1)	3.9 (1.1)	4.0 (1.1)	0.04
(95% CI)	(3.8, 4.0)	(3.5, 3.9)	(3.7, 4.1)	(3.8, 4.2)	
Change TC/HDL Ratio	-0.4 (0.9)	-0.1 (0.9)	-0.5 (0.9)	-0.5 (1.0)	0.00
(95% CI)	(-0.5, -0,3)	(-0.2, 0)	(-0.6, -0.4)	(-0.6, -0.4)	
% Change TC/HDL Ratio	-7.3 (21.6)	-0.6 (22.0)	-10.7 (20.0)	-10.6 (21.2)	0.00
(95% CI)	(-9.2, -5.4)	(-4.0, 2.8)	(-14.0, -7.4)	(-13.6, -7.6)	
