# Supplementary material for: High intensity lifestyle intervention and long-term impact on weight and clinical outcomes
Source: PLoS One. 2018 Apr 18;13(4):e0195794. doi: 10.1371/journal.pone.0195794 (PMC5905976; doi:10.1371/journal.pone.0195794)
Supplement: S5 Table — (PDF) [file pone.0195794.s007.pdf]

Supporting Information

S5 Table: Weight Loss Efficacy (mean, sd) by Status of Medication Use: Standard International Unit Metric System with 95% confidence interval

	No Medication at baseline		Medication at baseline		
	No medication at follow-up	Medication at follow-up	No medication at follow-up	Medication at follow-up	p-value
Cholesterol medication					
N (%)	295 (59.0)	13 (2.6)	43 (8.6)	149 (29.8)	
Baseline Weight kg	107.8 (26.3)	123.0 (34.3)	114.2 (25.6)	113.5 (31.3)	0.08
(95% CI)	(104.8, 110.8)	(102.3, 143.8)	(106.3, 122.0)	(108.4, 118.6)	
Change Weight kg	-21.0 (12.9)	-25.3 (15.5)	-29.4 (17.5)	-20.3 (14.7)	0.00
(95% CI)	(-22.5, -19.5)	(-34.6, -15.9)	(-34.8, -24.0)	(-22.7, -17.9)	
% Change Weight	-18.9 (9.1)	-19.7 (10.6)	-24.7 (10.7)	-17.2 (9.2)	0.00
(95% CI)	(-19.9, -17.9)	(-26.1, -13.3)	(-28.0, -21.5)	(-18.7, -15.7)	
Anti-hypertensive					
N (%)	253 (50.6)	12 (2.40)	79 (15.8)	156 (31.2)	
Baseline Weight kg	103.7 (23.0)	116.6 (27.4)	112.9 (23.2)	119.7 (34.6)	0.00
(95% CI)	(100.8, 106.5)	(99.3, 134.0)	(107.7, 118.1)	(114.3, 125.2)	
Change Weight kg	-20.0 (12.3)	-24.1 (13.7)	-26.3 (16.3)	-21.8 (15.4)	0.00
(95% CI)	(-21.5, -18.4)	(-32.8, -15.4)	(-29.9, -22.6)	(-24.3, -19.4)	
% Change Weight	-18.7 (9.2)	-20.1 (8.5)	-22.4 (10.7)	-17.4 (8.8)	0.00
(95% CI)	(-19.8, -17.5)	(-25.5, -14.7)	(-24.8, -20.0)	(-18.8, -16.0)	
Oral Anti-diabetics					
N, %	391 (85.4)	-	27 (5.90)	40 (8.73)	
Baseline Weight kg	107.2 (25.5)	-.	123.5 (26.1)	114.8 (37.9)	0.00
(95% CI)	(104.7, 109.8)		(113.2, 133.8)	(102.6, 126.9)	
Change Weight kg	-20.8 (12.5)	-.	-28.6 (18.6)	-21.0 (13.8)	0.08

(95% CI)	(-22.0, -19.5)		(-36.0, -21.2)	(-25.4, -16.6)	
% Change Weight	-18.8 (9.0)	-	-22.2 (12.5)	-17.5 (6.7)	0.35
(95% CI)	(-19.7, -17.9)		(-27.1, -17.2)	(-19.6, -15.3)	
